# Clinical and biochemical effects of a combination botanical product (ClearGuard™) for allergy: a pilot randomized double-blind placebo-controlled trial

**DOI:** 10.1186/1475-2891-7-20

**Published:** 2008-07-14

**Authors:** Jonathan Corren, Marc Lemay, Yumei Lin, Lisa Rozga, R Keith Randolph

**Affiliations:** 1 Allergy Research Foundation, Los Angeles, USA; 2 Supplement Product Development, Nutrilite Health Institute, Buena Park, USA; 3 Analytical Services, Alticor Inc, Ada, USA

## Abstract

**Background:**

Botanical products are frequently used for treatment of nasal allergy. Three of these substances, *Cinnamomum zeylanicum*, *Malpighia glabra*, and *Bidens pilosa*, have been shown to have a number of anti-allergic properties *in-vitro*. The current study was conducted to determine the effects of these combined ingredients upon the nasal response to allergen challenge in patients with seasonal allergic rhinitis.

**Methods:**

Twenty subjects were randomized to receive the combination botanical product, (CBP) 2 tablets three times a day, loratadine, 10 mg once a day in the morning, or placebo, using a randomized, double-blinded crossover design. Following 2 days of each treatment and during the third day of treatment, subjects underwent a nasal allergen challenge (NAC), in which nasal symptoms were assessed after each challenge dose and every 2 hours for 8 hours. Nasal lavage fluid was assessed for tryptase, prostaglandin D2, and leukotriene E4 concentrations and inflammatory cells.

**Results:**

Loratadine significantly reduced the total nasal symptom score during the NAC compared with placebo (P = 0.04) while the CBP did not. During the 8 hour period following NAC, loratadine and the CBP both reduced NSS compared with placebo (P = 0.034 and P = 0.029, respectively). Analysis of nasal lavage fluid demonstrated that the CBP prevented the increase in prostaglandin D2 release following NAC, while neither loratadine nor placebo had this effect. None of the treatments significantly affected tryptase or leukotriene E4 release or inflammatory cell infiltration.

**Conclusion:**

The CBP significantly reduced NSS during the 8 hours following NAC and marginally inhibited the release of prostaglandin D2 into nasal lavage fluid, suggesting potential clinical utility in patients with allergic rhinitis.

## Background

Recent surveys conducted in the United States reveal that a significant proportion of the population have utilized botanical products for the treatment of nasal allergy symptoms [1-3]. While many of these substances have been widely used throughout the world, there have been relatively few controlled clinical trials to support their use. Several botanicals, including Spanish needles (*Bidens pilosa*) [4,5] and cinnamon extract (*Cinnamomum cassia*)[6] have been reported to have significant effects upon a number of allergic processes, including but not limited to the inhibition of histamine release and synthesis of lipid-derived mediators. Based upon these prior data, a number of candidate substances were screened for their effects on basophil histamine release (data not shown). The results of the histamine release experiments suggested that three plant materials, including Spanish needles, cinnamon (*Cinnamomum zeylanicum*), and acerola (*Malpighia glabra*) might be most useful in inhibiting the acute effects of allergen exposure. The purpose of the current study was to determine the subjective (nasal symptoms) and objective (sneeze counts, peak expiratory flow, nasal lavage inflammatory mediator bioassays) effects of a combination of Spanish needles powder, cinnamon extract, and acerola extract, upon early and late-phase responses to nasal allergen challenge, compared with an oral H1 antihistamine and placebo, in patients with seasonal allergic rhinitis.

## Methods

### Study design

A randomized, double-blind, placebo-controlled, double-dummy, crossover, single-center clinical study was designed to assess the effivacy of ClearGuard™ (combination botanical product, CBP) for allergy symptoms. The CBP is composed of equal parts by weight Cinnamon (*Cinnamomum zeylanicum*) bark extract (4:1 ethanol and water extract), acerola (*Malphighia glabra*) fruit concentrate (concentrated by filtration and dehydration to a ratio of 1:9.5), and Spanish Needles (*Bidens pilosa*) leaf and stem dehydrated powder (CBP; ClearGuard™, Access Business Group LLC, Buena Park, CA). CBP treatment was 450 mg three times daily (given at approximately equal intervals throughout the day). Other treatments were loratadine (Claritin™)10 mg once a day in the morning; and placebo, three times daily.

The CBP and loratadine were in tablet form. Two different placebos were also provided to subjects, one in capsule form and one in tablet form. Study products were provided in tear-pouches containing individual servings labelled for order of consumption (first, second, or third dose of the day). All subjects took a combination of tablets and capsules at each dose, in a combination of active product or placebo as necessary to maintain blinding. No one having direct contact with the subjects had knowledge of the identity of the products.

Each treatment involved 3 doses a day for 3 days, with the third day of dosing occurring on the day of the nasal allergen challenge (NAC). Each study treatment was separated by a period of 3 to 7 days of no treatment and no nasal allergen challenge. Over a period of about three weeks, subjects were to be exposed to all three treatments in one of the following sequences: CBP, loratadine, Placebo (CLP sequence); loratadine, Placebo, and CBP (LPC sequence); loratadine, CBP, Placebo (LCP sequence); or Placebo, CBP, loratadine (PCL sequence). Random assignment to treatment sequence was effected by means of a randomization list provided by the Sponsor. The list featured twenty four-digit subject numbers in the first column, and a sequence name ("Sequence 1" through "4") in the second column. Subjects were sequentially assigned a subject number with its associated sequence as they were enrolled.

The study protocol was approved by Coast IRB (Colorado Springs, CO) and was conducted in compliance with the Declaration of Helskinki. Every subject gave signed informed consent before any study procedures were initiated. The study took place from July to August 2005 at the Allergy Research Foundation, Los Angeles, California.

### Subjects

Twenty adults, currently asymptomatic but with at least a 2 year history of seasonal allergic rhinitis during the spring, summer and/or fall seasons were recruited for the study. Subjects had to be non-smoking adults aged 18 to 65 years, and were required to have a positive skin prick test (mean wheal diameter 3 mm greater than saline control) to a standardized extract of Timothy grass (100,000 AU/ml; Greer Labs, Lenoir, NC). Subjects also had to be pollen or animal-dander allergy asymptomatic at the time of the study, and in good general health as determined by a medical exam and history, and standard serum biochemistry, hematology, urinalysis. Exclusion criteria included the current use of allergy relief medication; the use of dietary supplements such as vitamins, minerals, and herbals products or drinks within one week of the start of the study; a clinically significant history or presence of cancer, cardiovascular disease, endocrine, kidney, liver, lung, gastrointestinal, or metabolic disorder, any other chronic health condition such as diabetes identified from the findings of the interview. Subjects with nasal pathology other than seasonal allergy that resulted in significant nasal obstruction, with a recent history of viral upper respiratory infection or sinusitis 6 weeks before the study, or who drank greater than two alcoholic beverages a day on average were excluded, as were subjects who had participated in another clinical trial within 30 days of enrollment into the study, pregnant or lactating women, or women of child-bearing potential unwilling to use a medically approved form of birth control.

### Nasal Allergen Challenge

The nasal allergen challenge (NAC) procedure was performed using a spray bottle delivering 0.07 ml per spray. Diluent (NaCl, 0.9%, albumin, 0.3%), 2 sprays per nostril, was administered initially, followed by increasing concentration of allergen extract (50, 250, 1250 and 6250 AU/ml), 2 sprays per nostril, given every 10 minutes. Both nostrils were sprayed at each allergy concentration (two sprays per nostril, for a total of four sprays per concentration). Not all subjects underwent the full dose-response due to maximum possible response before reaching the full dose (6250 AU/ml). Patients left the clinic after all procedures were conducted during the NAC, and were not allowed to use any symptomatic treatment over the next 24 hours.

### Clinical assessments

Ten minutes after diluent and each dose of allergen, and 2, 4, 6 and 8 h after the completion of the NAC, patients rated their nasal and ocular symptoms as follows: absolute sneeze count per two hour period; score on the Nasal Symptom Scale, comprising the components sneezes (in last two hours: 3 sneezes = 1, > 5 sneezes = 2); rhinorrhea (anterior discharge = 1, postnasal drip = 1, both = 3); nasal blockage (breathes freely = 0, breathes with difficulty = 1, one nostril blocked = 2, both nostrils blocked = 3); pruritis (nasal = 1, palate or ear = 1); conjunctivitis (any ocular itch = 1). Nasal inspiratory peak flow was also measured at all of the same time points, in triplicate, and the best value was recorded.

### Nasal lavage

Nasal lavage was performed by instilling and collecting 5 ml of buffered nasal saline per nostril prior to NAC, 10 minutes after the completion of NAC, and 24 hours later. Prostaglandin D2, leukotriene E4, and tryptase were measured in lavage fluid at each of the above time points. Cell counts were performed on undiluted lavage fluid and differential counts were performed on Wright stains of lavage samples centrifuged at 4°C for 10 min at 400 g, prior to and 24 hours after each nasal challenge.

### Safety monitoring

Incidence and severity of adverse events, as well as any changes from baseline in laboratory values including complete blood count and differential, chemistry panel, and urinalysis, were assessed at the end of each treatment period.

### Statistics

A sample size of 20 (all subjects exposed to all treatments in a crossover design) provided 80% power at the alpha level of 0.05 to detect a mean difference of 0.5 points on the Nasal Symptoms Scale, which corresponds to the smallest clinically meaningful difference in allergy treatment on an composite scale like the NSS [7]. The primary efficacy measure was change from baseline NSS scores at each time point, as well as total NSS scores. Secondary outcome measures included nasal inspiratory peak flow rate, PGD2, leukotriene E4, tryptase, total cell count, and percent eosinophils, which were assessed at both discrete time points and using the average across time points during the early (acute challenge) and late (hours 2–8) phases comparing the 2 active treatments with placebo using one-tailed paired t-tests. Results are expressed as group means with standard errors of the means. An alpha value of P < 0.05 following a paired *t *test was considered significant. Due to the pilot nature of this study, no corrections were made for multiple comparisons. All randomized subjects were included in the intent-to-treat (ITT) population. The ITT population was defined as all subjects were took at least one dose of study product followed by the NAC, and then at least one dose of another study product followed by the NAC. The safety population included all subjects who were randomized, took at least one dose of study product, and who returned to provide any safety follow-up evaluation (physical examination, laboratory tests, subjective reports).

## Results

### Subjects

Twenty subjects (11 women) with a mean age of 38 years gave their informed consent and were screened for participation in this study. Seventeen subjects completed all treatments (loratadine, CBP, placebo); 3 subjects who were did not complete all test procedures were excluded from pairwise analyses on a per-comparison basis (Table 1). Due to error during allocation to treatment sequences, five subjects were given the CBP (C), Placebo (P) and loratadine (L) treatments in sequences other than intended, as follows [sequence: *intended n*/*actual n*]: CLP: *5/2*; LPC: *5/4*; LCP: *5/4*; PCL: *5/4*; PCL: *0/5*.

**Table 1:** Demographics of the study population

No. of subjects enrolled	20
No. of subjects completing all treatment conditions	16
Mean age (SD) (y)	38.2 (3.34)
Mean height (SD) (in)	64.7 (3.56)
Mean weight (SD) (lb)	153.5 (31.57)
Sex (%)	
Male	45
Female	55

### Clinical response

Because some subjects reported the highest possible nasal symptom scores at Allergen Units/ml lower than the maximum, not all subjects were exposed to the two highest challenges. There were no treatment differences in the number of subjects who were unable to tolerate the highest allergen challenges: Of 20 subjects per allergen dose, all subjects were able to tolerate the 50 AU/ml and 250 AU/ml challenges; but for each treatment, four subjects were not exposed to the 1250 AU/ml challenge, and five subjects were not exposed to the 6250 AU/ml challenge.

**Figure 1:**
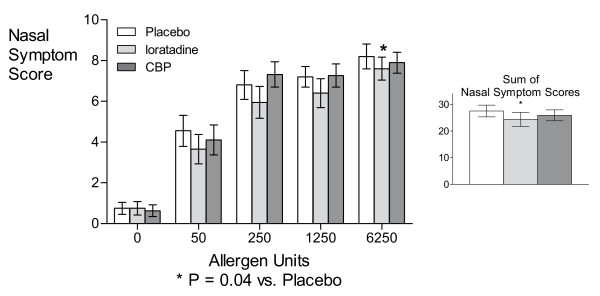
**Nasal Symptom Scale scores by allergen challenge dose and treatment**. Not all subjects underwent the full dose-response due to maximum possible response before reaching the full dose (6250 AU/ml). The number of subjects tested at 0, 50, 250, 1250, and 6250 AU/ml were respectively 20, 20, 20, 15, and 13 in the Placebo treatment condition; 20, 20, 20, 14, and 13 in the loratadine treatment condition; and 19, 19, 19, 15, and 13 in the CBP treatment condition.

Total nasal symptom scores measured immediately after each dose of allergen were significantly suppressed by loratadine at the highest dose of allergen (6250 AU) (P = 0.04; Figure 1), as well as overall (P = 0.04; Figure 1, inset) expressed as the sum of nasal symptoms experienced over the NAC compared with placebo, while the CBP had no effects on nasal symptoms at these time points.

**Figure 2:**
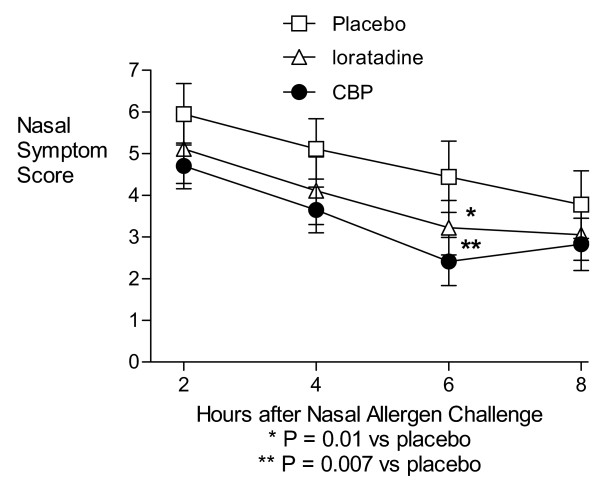
**Nasal Symptom Scale scores by time and treatment**. Results from an 8-hour period of at-home ratings by subjects following the laboratory-based Nasal Allergen Challenge. Because not all subjects underwent the full dose-response in the laboratory, the results above represent symptoms following a maximum dose of 250, 1250, or 6250 AU/ml from, respectively, 5, 2, and 13 subjects in the Placebo treatment condition; 6, 1, and 13 subjects in the loratadine treatment condition; and 4, 2, and 13 subjects in the CBP treatment condition.

**Figure 3:**
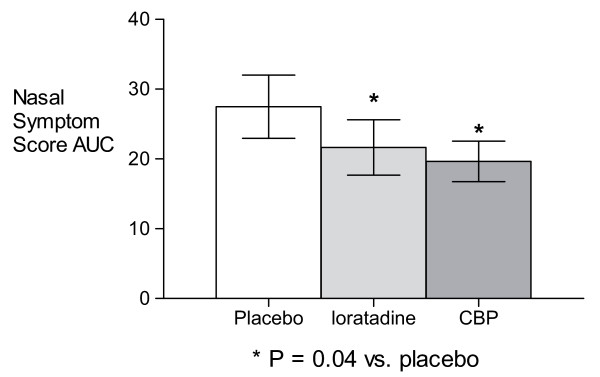
Nasal Symptom Scale scores Areas Under the Curve, 2–8 h after Nasal Allergen Challenge.

During the 8 hour observation period following the NAC, treatment with both the CBP and loratadine resulted in significantly lower mean nasal symptom scores than placebo at the 6 hour time-point (P = 0.007 and 0.01, respectively; Figure 2) and for hours 2–8 area-under-curve (P = 0.04 for both treatments; Figure 3) compared with placebo. The CBP and loratadine were not significantly different from each other at any time point or for the area-under-curve analysis. There were no differences in peak expiratory flow rates at any time with any treatment.

Compared with placebo, the CBP reduced sneezing at 6 hours (P = 0.04) (Figure 4) as well as for the AUC analysis (P = 0.04), while loratadine led to significantly fewer sneezes compared to placebo at 6 and 8 hours (P = 0.02 and 0.03) but not for the AUC analysis. Other individual symptom scores were not significantly affected by either active treatment (Table 2).

**Table 2:** Nasal Symptom Scale scores and sneeze counts during late response phase

Comparison	Measure	Hours after Nasal Allergen Challenge	Treatment	Mean	N	SD	t	P (1-tailed)	
Loratadine- Placebo	Nasal Symptoms Score	2	Loratadine	5.1	18	3.5	-1.10	0.143	
			Placebo	5.9	18	3.1			
		4	Loratadine	4.1	18	3.4	-1.43	0.086	#
			Placebo	5.1	18	3.1			
		6	Loratadine	3.2	18	2.8	-2.57	0.010	**
			Placebo	4.4	18	3.6			
		8	Loratadine	3.1	18	2.6	-1.05	0.153	
			Placebo	3.8	18	3.4			
		AUC	Loratadine	15.5	18	10.7	-1.82	0.044	*
			Placebo	19.3	18	12.6			
	Sneeze count	2	Loratadine	2.2	17	3.0	-1.25	0.114	
			Placebo	3.8	17	5.5			
		4	Loratadine	2.6	17	4.0	-0.52	0.306	
			Placebo	3.2	17	4.5			
		6	Loratadine	1.4	17	2.3	-2.22	0.021	*
			Placebo	3.6	17	5.8			
		8	Loratadine	1.2	17	1.6	-1.89	0.038	*
			Placebo	2.7	17	3.8			
		AUC	Loratadine	7.5	17	7.8	-1.79	0.047	*
			Placebo	13.2	17	17.7			
Placebo-CBP	Nasal Symptoms Score	2	Placebo	5.8	17	3.1	1.39	0.091	#
			CBP	4.7	17	2.3			
		4	Placebo	4.9	17	3.1	1.71	0.053	#
			CBP	3.6	17	2.3			
		6	Placebo	4.2	17	3.5	2.74	0.007	**
			CBP	2.4	17	2.4			
		8	Placebo	3.5	17	3.3	0.90	0.190	
			CBP	2.8	17	2.6			
		AUC	Placebo	18.4	17	12.4	1.86	0.041	*
			CBP	13.6	17	8.0			
	Sneeze count	2	Placebo	3.2	17	5.2	1.57	0.068	#
			CBP	1.2	17	1.6			
		4	Placebo	2.7	17	4.4	1.72	0.052	#
			CBP	1.2	17	1.9			
		6	Placebo	3.4	17	5.8	1.93	0.036	*
			CBP	0.7	17	1.0			
		8	Placebo	2.3	17	3.7	0.90	0.191	
			CBP	1.5	17	2.3			
		AUC	Placebo	11.5	17	17.2	1.76	0.049	*
			CBP	4.6	17	3.9			
Loratadine- CBP	Nasal Symptoms Score	2	Loratadine	4.8	17	3.4	0.14	0.447	
			CBP	4.7	17	2.3			
		4	Loratadine	3.9	17	3.4	0.28	0.393	
			CBP	3.6	17	2.3			
		6	Loratadine	3.1	17	2.8	1.43	0.086	#
			CBP	2.4	17	2.4			
		8	Loratadine	2.9	17	2.6	0.20	0.421	
			CBP	2.8	17	2.6			
		AUC	Loratadine	14.7	17	10.5	0.51	0.308	
			CBP	13.6	17	8.0			
	Sneeze count	2	Loratadine	1.8	16	2.6	0.88	0.197	
			CBP	1.3	16	1.6			
		4	Loratadine	2.4	16	4.0	1.09	0.145	
			CBP	1.3	16	1.9			
		6	Loratadine	1.3	16	2.4	1.07	0.151	
			CBP	0.7	16	1.0			
		8	Loratadine	1.1	16	1.5	-0.55	0.295	
			CBP	1.4	16	2.3			
		AUC	Loratadine	6.6	16	7.1	1.18	0.128	
			CBP	4.7	16	4.1			

# P < 0.10, * P < 0.05, ** P ≤ 0.01

**Figure 4:**
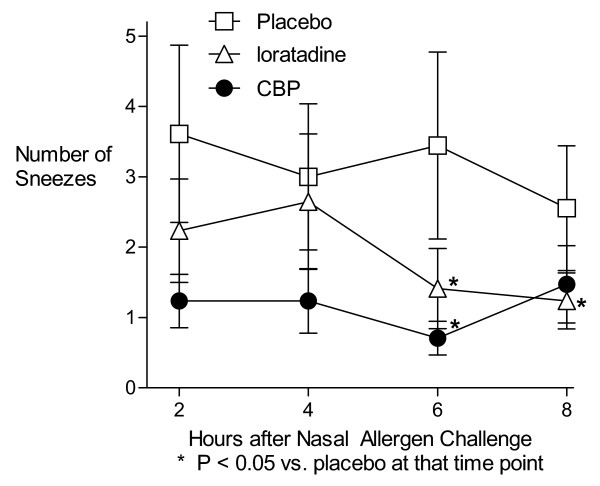
Sneezes per 2-hour period after Nasal Allergen Challenge.

### Analysis of nasal fluid mediators and inflammatory cells

**Figure 5:**
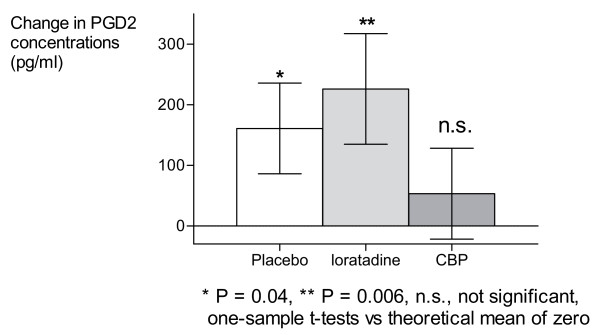
Change in nasal lavage fluid PGD2 concentrations after Nasal Allergen Challenge.

Analysis of mediator concentrations in nasal lavage fluid following NAC demonstrated that the CBP prevented the rise in prostaglandin D2 from baseline levels (P = 0.62), while neither placebo nor loratadine treatments blocked these increases (P = 0.04 and 0.006 respectively; Figure 5). However, there were no significant intergroup differences for this parameter. Neither active treatment significantly affected tryptase or leukotriene E4 release or inflammatory cell infiltration (data not shown) compared to placebo.

### Adverse events

Blood chemistries, hematology, and urinalysis were performed before and after each three-day period of dosing. Review of laboratory findings and subjective adverse event reports revealed no untoward signs or symptoms associated with consumption of any of the products during these three-day dosing periods.

## Discussion

The objective of the current study was to determine the effects of the botanical product, compared with placebo and loratadine, upon the clinical and inflammatory responses to nasal allergen provocation. We demonstrated that the CBP significantly reduced nasal symptoms during hours 2 through 8 following the challenge and blocked the post-allergen rise in prostaglandin D2. Subjectively, the magnitude of the difference between placebo and the CBP was about 1.5 points on the 11-point Nasal Symptoms Scale (NSS), which is greater than The smallest clinically meaningful difference in allergy treatment effects is about a 0.5 score step on an composite scale like the NSS [7]. Thus, the clinical results with the CBP were not only statistically significant but also clinically meaningful, a conclusion also supported by the comparison to loratadine, which was associated with symptom relief greater than placebo but not significantly different from the CBP.

Our study had limitations. In particular, the study population was small, and without having any knowledge of the pharmacokinetics of the botanical product, it is difficult to say whether a washout period of 3 to 7 days between treatments was sufficient to prevent carry-over effects. In addition, while our data indicate significant differences between the experimental therapy and placebo, it is important to note that these effects were statistically assessed using one-tailed paired t-tests without corrections for multiple comparisons, as befits the pilot nature of this study. Finally, the generalizability of these laboratory-based results could be investigated in further laboratory-based or native disease studies.

While the combination was shown to have selected effects upon allergic pathophysiology, we are unable to determine which of the three components contributed most to these effects. Our choice of botanical substances for this study was based upon prior *in-vitro *experiments which demonstrated potentially beneficial effects with either the individual components used in the CBP or phytochemicals derived from these substances. A species of cinnamon related to that found in the CBP, *Cinnamomum cassia*, has been found to inhibit complement-dependent allergic reaction by reducing immunological hemolysis, chemotactic migration of neutrophils, and the generation of chemotactic factors by mast cells in response to complement-activated serum [6]. Acerola contains vitamin C, which has been shown to reduce concentrations of histamine [8,9]. Spanish Needles (*Bidens pilosa*) has also been found to have anti-allergic effects on mast cells and synthesis of several inflammatory mediators [10]. Quercetin, a flavonoid found in Spanish Needles [11,12], has been shown to stabilize mast cells and basophils, decrease leukotriene synthesis and reduce the release of histamine and other mediators [13]. Spanish Needles also contains ethyl caffeate which has been shown *in vitro *to possess a variety of anti-inflammatory effects [4,11]. Spanish Needles has been shown to inhibit nuclear transcription factor kappaB and its downstream inflammatory mediators *in vitro *[4,5]. The actual active ingredients are unknown, however, which is typical for botanical products [14]. For example, St-John's Wort, a single-ingredient botanical for depression which was once thought to possess a single active molecule, is now known to possess several active constituents [5], and its anti-depressant activity can only be ascribed to the total extract, not to any specific molecule or combination of molecules [15]. While this general state of affairs accords well with the ancient concept of botanical therapeutics, whereby the efficacy of any botanical – and its possible advantage over a traditional single-compound pharmaceutical – stems from its complex action against multiple pharmacological targets [16], this poses manufacturing and quality control challenges [17]. The solution adopted by painstaking manufacturers in the industry entails a combination of analytical techniques and bioassays to ensure product standardization [18,19]. The manufacture of the present CBP involves testing for the marker substances chlorogenic acid and cynarin for the Spanish Needles powder, cinnamic acid for the cinnamon extract, and ascorbic acid for the acerola extract, all verified by HPLC; as well as total polyphenols for the finished tablet, verified by UV/Vis spectroscopy. Microbiological monitoring is also involved in quality control in according with Good Manufacturing Practices.

While prior experiments using the individual components or relevant isolated phytochemicals had particularly prominent effects on histamine release and leukotriene synthesis, in the current study the combination of the three botanical products had no detectable effects on tryptase or leukotriene C4 concentrations in nasal lavage fluid nor any effect on the acute clinical response to allergen challenge. A possible explanation for this discrepancy between previous *in vitro *data and the current results in allergic patients may relate to pharmacokinetic issues, including the attainment of adequate tissue levels of the active ingredients, or may reflect biologic differences between basophil or animal mast cell systems and human mucosal mast cells [20].

While the immediate, or early nasal response to allergen provocation was not affected by the botanical preparation, we did note a significant reduction in nasal symptom scores during the 8 hour time period following nasal challenge. The most likely explanation for this was the apparent reduction in increased PGD2 secretion in the CBP-treatment condition, compared to the loratadine- or Placebo-treatment conditions, noted immediately after allergen exposure. To our knowledge, no prior studies have examined the acute effects of PGD2 administration upon nasal symptoms or function in humans. However, injection of PGD2 into the skin of nonallergic patients has been shown to cause induration which lasted up to 6 hours [21]. In addition, one prior clinical trial in patients with seasonal allergic rhinitis demonstrated that the addition of an oral cyclooxygenase-1 inhibitor (naproxyn sodium) to a combination of oral H1-antihistamine plus decongestant (chlorpheniramine plus pseudoephedrine) augmented nasal symptom control significantly [22]. Both of these experimental findings suggest the role of PGD2 in eliciting tissue inflammation and the potential importance of blocking its effects in patients with allergic nasal disease.

Consistent with our prior knowledge of these phytochemicals, no clinical or laboratory adverse events were noted during this short-term trial. Although toxicity is considered unlikely, longer-term studies with the botanical product would help to clarify its place in the clinician's armamentarium for treating patients with persistent symptoms.

The medical speciality of allergy and immunology has recently seen an increase in the use of complementary and alternative medicine by both patients and physicians [2]. Rational and effective use of such therapies requires controlled clinical trials to demonstrate safety and efficacy. However, because of the multifarious nature of botanicals compared to pure single pharmaceuticals, some adjustments are required to principles of Good Clinical Practice [14]. In the present study, these are centered on the identity of the active ingredients in the CBP, which are unknown.

## Conclusion

In this study, a dietary supplement composed of three botanical ingredients was shown to safe and effective in a well-recognized model of anti-allergic action. Its symptom-reduction action was comparable to the positive control treatment loratadine. The CBP reduced nasal symptoms occurring 2 to 8 hours after nasal allergen challenge and attenuated increases in PGD2 collected from nasal lavage fluid. These clinical attributes hold promise as a potential therapy for allergic rhinitis.

## Competing interests

JC received payment from the Sponsor for participation in the clinical trial. The other four authors are employees of the Sponsor.

## Authors' contributions

JC conceived of the study, and participated in its design and coordination and helped to draft the manuscript. ML participated in the design of the study and its coordination, performed the statistical analyses, and helped draft the manuscript. YL, LR and RKR participated in the design of the study, and helped draft the manuscript. All authors read and approved the final manuscript.
